# Color difference between the vita classical shade guide and composite veneers using the dual-layer technique

**DOI:** 10.4317/jced.59759

**Published:** 2022-08-01

**Authors:** Franciele Floriani, Bryce-Arielle Brandfon, Nathalie J. Sawczuk, Guilherme-Carpena Lopes, Mateus-Garcia Rocha, Dayane Oliveira

**Affiliations:** 1 PhD. Candidate at the Federal University of Santa Catarina, Florianópolis, Brazil; 2 Research Volunteer, Department of Restorative Dental Sciences, School of Dentistry, University of Florida, Gainesville, FL, United States; 3 Professor, Department of Operative Dentistry, Federal University of Santa Catarina, Florianópolis, Brazil; 4 Clinical Assistant Professor, Center for Dental Biomaterials, Department of Restorative Dental Sciences, College of Dentistry, University of Florida, Gainesville, FL, United States

## Abstract

**Background:**

The purpose of this in vitro study was to evaluate the color difference between the Vita Classical Shade Guide and composite veneers using the dual-layer technique.

**Material and Methods:**

Thirty samples were fabricated using a custom-made mold (Easy Layering Shade Guide Kit, 3M) using two resin composites: Filtek Supreme Ultra (3M); and Estelite Omega (Tokuyama) (n=3). The composite veneers were made by layering the different enamel and body or dentin shades from each composite. The color measurements were taken using a spectrophotometer (Vita Easyshade V®, Vita Zahnfabrik). The ΔE00 between the Vita Classical Shade Guide (Vita Zahnfabrik) and the composite veneers were calculated using the CIEDE2000 formula.

**Results:**

For the composite veneers using Filtek Supreme Ultra, the best match for A1 Vita shade was achieved layering either EA1 with DA2 or DA3; EA2 with DA1 or DA2 (ΔE00= 1.53 ~ 1.96 ± 0.4). For A2 Vita shade the best match would be EA3 with DA3 or EA3 with DA2 (ΔE00= 1.40 ~ 1.85 ± 0.1); or for A3 Vita shade the best match would be EA3 with DA2 2.50±(0.6). For the composite veneers using Estelite Omega, the were no best match for neither A1, A2 or A3 Vita shade (ΔE00> 2.5).

**Conclusions:**

The combination of enamel and dentin shades from Filtek Supreme Ultra provided acceptable color match for A1, A2 and A3 shades from the Vita Shade Guide, while Estelite Omega did not provide acceptable color match for any of the Vita Shade Guide standard shades tested.

** Key words:**Color, color matching, optical properties, resin composite, layering.

## Introduction

Color is generally described based on the Munsell System and the International Commission on Illumination (CIE) color/order system (1). According to the Munsell system (1), color has three dimensions: hue, value, and chroma. Hue is how the color is distinguished from another color (red, green, blue, yellow), chroma is the intensity or saturation of the hue, and value is defined as the quantity of light an object reflects when compared to a pure white diffuser and black absorber (amount of black and white) (1,2).

The most common method to select color in dentistry is the visual comparison using shade guides. Although it is a subjective method, it can be precise depending on the clinician’s experience (3). However, most composite manufacturers do not have their custom-made shade guides for direct restorations. Instead, the most common practice is to use the Vita Classical Shade Guide as a standard (4). The main concern is that there is not a standard resin composite shade nomenclature (5). Although composite manufacturers name their shades similar to the Vita Classical Shade Guide nomenclature, it does not necessarily correlate with the Vita shades (6,7). For example, 58% of dental educators complain about the mismatch between the shade guides and the resin composite (5). This discrepancy was tentatively explained by the fact that the shade guide is not made with the same material and thickness as the composite restoration (5,6). Thus, it becomes even more challenging to select and match color for direct restorations (8).

Besides that, most shade guides do not demonstrate adequate optical properties due to the enamel and dentin layer not having the proper thickness of natural teeth (7). To achieve esthetics in restorations, the optical properties of both the restorative materials and natural teeth should match (8). Resin composite’s optical properties are, in fact, strongly influenced by the composite-layering technique, which allow clinicians to emulate natural teeth biological appearance, producing more vital-looking restorations (9). However, with this technique, the shade for the final layer of the restoration is rarely predicTable (6,7).

In addition, manufacturers generally do not determine the color thickness of the final enamel layer needed to produce a specific color (8,10). Maintaining the proper range of thicknesses in each layer is necessary for achieving a desirable shade, as changes in the thickness of each layer can significantly alter the final shade of the restoration (8,11). Therefore, this *in vitro* research aimed to evaluate the color matching when layering enamel and dentin shades using two resin composites in comparison to the Vita Classical Shade Guide standard shades. The null hypothesis was that there would be no difference in color between the Vita Classical Shade Guide and composite veneers using the dual-layer technique with their respective enamel/dentin shades.

## Material and Methods

-Composite Veneers using dual-layer technique

Two commercially resin-based composite were used in this study: Filtek Supreme Ultra (3M, St. Paul, MN, United States) and Estelite Omega (Tokuyama, Tokyo, Japan). The composites veeners were made layering the following enamel and dentin/body shades together: EA1/DA1; EA1/DA2; EA1/DA3; EA1/DA4; EA1/BA1; EA1/BA2; EA1/BA3; EA2/DA1; EA2/DA2; EA2/DA3; EA2/DA4; EA2/BA1; EA2/BA2; EA2/BA3; EA3/DA1; EA3/DA2; EA3/DA3; EA3/DA4; EA3/BA1; EA3/BA2; EA3/BA3.

**Figure 1:**
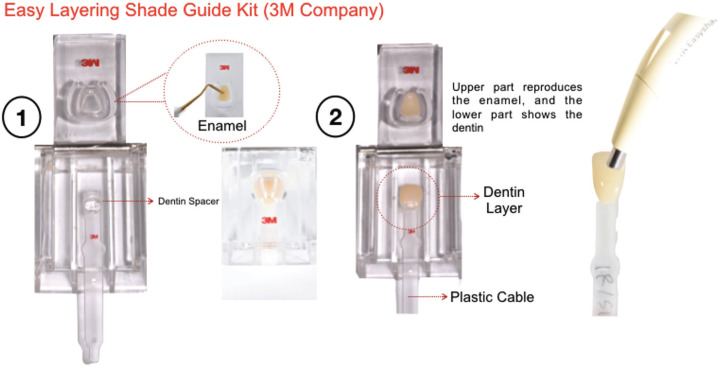
Composite Veeners step-by-step using the Easy Layering Shade Guide Kit.

All composite veneers were made using a custom matrix (Easy Layering Shade Guide Kit, 3M, St Paul, MN, United States) with standardized enamel and dentin layer thickness (Fig. 1). First, the enamel shade layer was placed, in which a standardized thickness of 1.1 mm was obtained by using a dentin spacer, as illustrated in Figure 1 (3,4). The enamel shade was light-cured for 20 seconds from the buccal side and 20 seconds from the lingual side (Valo Cordless, 1000 mW/cm2, Ultradent®, South Jordan, UT, United States) (12,13,14). Then, after removing the dentin spacer, the dentin layer was applied right above the cured enamel layer, with a transparent plastic cable attached in the back. The overall dentin thickness created by the dentin spacer was 1.5 mm in the middle and cervical thirds and 0.4 mm in the incisal third (3,4). The dentin layer was light-cured, following the same protocol described for the enamel layer (3,4).

-Color Measurements

The color was measured according to the CIE L*a*b* color scale relative to the standard illuminant D65 (Macbeth Judge II, X-Rite, Grand Rapids, MI, USA) over a white background using a spectrophotometer (Vita Easyshade V®, Vita Zahnfabrik, Bad Sackingen, Germany) (15). The color coordinate “L*” is an achromatic coordinate and refers to the lightness ranging from black (0) to white (100) (15,16). The coordinate “a*” is a chromatic coordinate that represents the green-red axis, in which negative values indicate green and positive values indicate red hue/chromas. The coordinate “b*” is also a chromatic coordinate that represents the blue-yellow axis, in which negative values indicate blue and positive values indicate yellow hue/chromas (15,17).

A Vita Classic shade guide (Vita Zahnfabrik, Bad Sackingen, Germany) was used as a gold standard for the standard shades A1, A2 and A3 (4). The color difference between the composite veneers and the Vita shade guide standard shades was calculated using the CIEDE2000 formula.

∆E00 = [(∆L/kL.SL)2 + (∆C/kC.SC)2 + (∆H/kH.SH)2 + RT.(∆C/kC.SC).( ∆H/kH.SH)]0.5 

Where, ∆L, ∆C and ∆H are the differences in lightness, chroma and hue, and RT is a function (the rotation function) that accounts for the interaction between chroma and hue differences in the blue region (16,17). The weighting functions, SL, SC, and SH are used to adjust the total color difference for variation in the location of the color difference pair in the L, a, and b coordinates. The parametric factors KL, KC, and KH, are correction terms for the experimental conditions, which were set to 1.

-Statistical Analysis

For the statistical analysis, data were collected and submitted to a two-way analysis of variance and Tukey’s test. These tests were used to assess the mean differences between the Vita Shade Guide standard shades A1, A2 and A3 and the composite veneers. The ΔE00 higher than 2.5 was considered statistically different (α=0.05) (18,19). A power analysis was conducted to determine sample size to provided a power of at least 0.8 at a significance level of 0.05 (β = 0.2).

## Results

Table 1 describes the ΔE00 values between the different enamel/dentin shades of the Filtek Supreme Ultra and the Vita Classical Shade Guide standard shades. The results show that for the A1 shade, the best match would be layering EA2 with DA1 or DA2 (ΔE00= 1.53 ± 0.8 and ΔE00= 1.83 ± 0.4, respectively), but layering EA1 with DA2 or DA3 would still provide an accepTable color match (ΔE00= 1.92 ± 0.3 and ΔE00= 1.96 ± 0.4, respectively). For the A2 shade, the best match would be layering EA2 with DA3 (ΔE00= 2.00 ± 0.3) or EA3 with DA3, DA2 or DA1 (ΔE00= 1.40 ± 0.4, ΔE00= 1.85 ±0.3 and ΔE00= 2.08 ± 0.3, respectively). For the A3 shade, the acceptable color match was EA3 with DA2 2.50±(0.6); all others different enamel and dentin combinations provided a ΔE00 > 2.5.

**Table 1:**
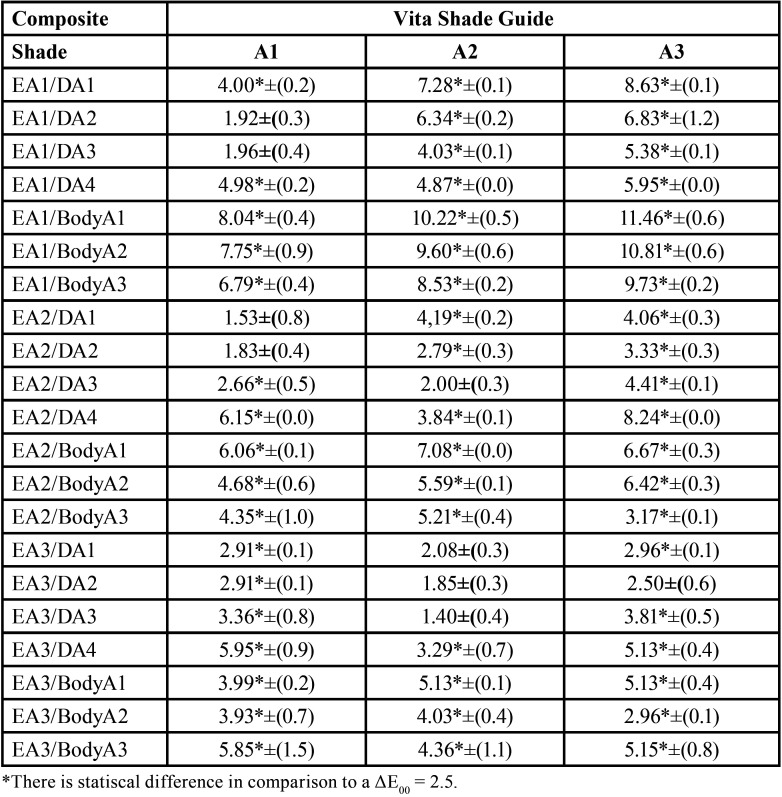
Color difference between Filtek Supreme Ultra enamel/dentin shades and the Vita shade guide standard shades.

Table 2 describes the ΔE00 values between the different enamel/dentin shades of the Estelite Omega and the Vita shade guide standard shades. The results show that there was not a satisfactory color match for any of the Vita Shade Guide standard shades. All enamel/dentin shades provided a ΔE00> 2.5 when compared with the Vita Shade Guide standard shades tested.

**Table 2:**
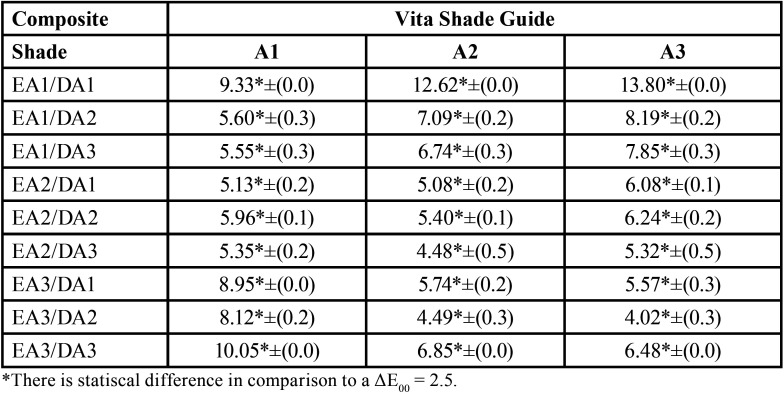
Color difference between Estelite Omega enamel/dentin shades and the Vita shade guide standard shades.

## Discussion

This study aimed to evaluate the color matching when layering different enamel and dentin composite shades and the Vita Classical Shade Guide standard shades A1, A2 and A3. The ΔE00 values between the Vita Classical Shade Guide shades and the enamel/dentin composite shades ranged from ΔE00= 1.40 ~ 11.46 ± 0.2 for Filtek Supreme Ultra and from ΔE00= 4.02 ~ 13.80 ± 0.3 for Estelite Omega. Our results agree with the *in vitro* study by Ferraris *et al*. (2014) that changes in enamel layering can result in entirely different values of chroma, hue, translucency, and opalescence (11).

Although there was an extensive range in the color differences between the different enamel and dentin shades layered and the Vita Classical Shade Guide standard shade goal, many of these differences may not be clinically visible. Waller *et al*. (2000) (18) analyzed the perceptibility and acceptability of color differences of a single-tooth implant. In which, dentists perceived no color differences at the restoration level up to a ΔE00 of 2.5 (18). Khashayar *et al*. (2014) (20) showed that the color difference establishes an accepTable shade or how much the observer perceives the color difference up to the limit of acceptability, and this value can vary between 2.0 and 4.0. In this study, only a few layered composites matched the keyed Vita Shade Guide standard shade. Out of the 163 combinations, 14 (8.58%) resulted in ΔE00 below the 2.5 clinically perceptible limit. The Filtek Supreme Ultra presented better results than the Estelite Omega when matching A1, A2 and A3 shades from the Vita Shade Guide.

As it can be observed in Table 1, the results allow different clinical reflections, pertinently to the aims of the current study. It was expected for A1 Vita Classical Shade Guide, that the composite veneers with EA1/DA1 would represent a more approximate value to A1 than EA1/DA2. Similarly, the A2 standard shade in the Vita Classical Shade Guide was closer to the combination of EA2 and DA3 (ΔE00 = 2.00 ± 0.3) than EA2 and DA2 (ΔE00 = 2.79 ± 0.3). The recommended color combinations of enamel/dentin layering shades were not necessarily the best matches compared to the respective shade of Vita -Classical Shade Guide standard.

Moreover, the results showed in Table 2 showed that Estelite Omega had no satisfactory color match for any of the Vita Shade Guide standard shades tested. This proves that although composite manufacturers name their shades similar to the Vita Classical Shade Guide nomenclature, it does not necessarily correlate with the Vita shades (6,7). Still, this can be an excellent composite to mimic lighter B-shades, but not darker A-shades. Therefore, the tested hypothesis that there would be no difference in color when layering enamel/dentin shades that correspond with the Vita Classical Shade Guide shades was rejected.

The final color appearance of a composite restoration depends on many factors, such as the composition of the composite itself (20), composite’s thickness according to the substrate’s color underneath it (20,21), pigment amount and type are the main contributory factors for the hue and the chroma of the final shade of the composite (22) Filtek Supreme Ultra contains a combination of silica (20 nm) and zirconia (4-11 nm) as filler particles with clusters formation ranging from 0.6 to 20 microns. The amount of filler particles ranges from 72.5% by weight (55.5% by volume) for translucent shades to 78.5% by weight (63.3% by volume) for opaque shades (22). The Estelite Omega contains spherical filler particles with an average particle size of 200 nanometers. These results agree with the literature that the layering technique decreases translucency with the change in the chroma of the dentin layer (12). Moreover, the amount of filler directly affects the translucency and lightness of the composites (11). Although the composition of the composite can explain an abundance of information, manufacturers do not fully disclose their composites’ composition. Indeed, it is known that the composition of composites from different manufactures varies greatly (23,24).

A defining limitation of this study is that only one thickness of enamel layer at 1.1 mm was evaluated. However, it is known the enamel thickness of anterior teeth only vary between ~0.8 mm and ~1.0 mm (25). This study considered the thickness of 1.0 mm as anterior teeth require more esthetic attention to color matching than other teeth. Moreover, color matching in Dentistry has always been a concerning issue. It is also worthwhile to mention that the range of shades in the shade guides is not consistent with the range of shades in natural teeth (3,7). Dental shade guides typically contain a limited selection of colors compared with those found in human teeth (18). Thus, dentists can achieve better color matching by directly choosing the enamel and dentin shades according to the tooth’s natural enamel and dentin shades to be restored. Further studies are still needed to evaluate further the correlation between layering techniques using other composites and other Vita Shade guide standard shades.

## Conclusions

Within the limitations of this *in vitro* study, this study showed that composite shades do not directly correlate to the Vita Classical Shade Guide shades. Still, it was possible to combine different enamel and dentin shades from Filtek Supreme Ultra to provide accepTable color match for A1, A2 and A3 shades from the Vita Shade Guide. However, Estelite Omega did not provide accepTable color match for any of these Vita Shade Guide standard shades.
